# A Modified Spectroscopic Approach for the Real-Time Detection of Pollen and Fungal Spores at a Semi-Urban Site Using the WIBS-4+, Part I

**DOI:** 10.3390/s22228747

**Published:** 2022-11-12

**Authors:** Emma Markey, Jerry Hourihane Clancy, Moisés Martínez-Bracero, Finnian Neeson, Roland Sarda-Estève, Dominique Baisnée, Eoin J. McGillicuddy, Gavin Sewell, David J. O’Connor

**Affiliations:** 1 School of Chemical Sciences, Dublin City University, D09 E432 Dublin, Ireland; 2 Laboratoire des Sciences du Climat et de l’Environnement (LSCE), CNRS-CEA-UVSQ, 91191 Saint-Aubin, France; 3 School of Chemical and Pharmaceutical Sciences, Technological University Dublin, D07 H6K8 Dublin, Ireland

**Keywords:** real-time, spore, pollen

## Abstract

The real-time monitoring of primary biological aerosol particles (PBAP) such as pollen and fungal spores has received much attention in recent years as a result of their health and climatic effects. In this study, the Wideband Integrated Bioaerosol Sensor (WIBS) 4+ model was evaluated for its ability to sample and detect ambient fungal spore and pollen concentrations, compared to the traditional Hirst volumetric method. Although the determination of total pollen and fungal spore ambient concentrations are of interest, the selective detection of individual pollen/fungal spore types are often of greater allergenic/agricultural concern. To aid in this endeavour, modifications were made to the WIBS-4 instrument to target chlorophyll fluorescence. Two additional fluorescence channels (FL4 and FL5 channels) were combined with the standard WIBS channels (FL1, FL2, FL3). The purpose of this modification is to help discriminate between grass and herb pollen from other pollen. The WIBS-4+ was able to successfully detect and differentiate between different bioaerosol classes. The addition of the FL4 and FL5 channels also allowed for the improved differentiation between tree (R^2^ = 0.8), herbaceous (R^2^ = 0.6) and grass (R^2^ = 0.4) pollen and fungal spores (R^2^ = 0.8). Both grass and herbaceous pollen types showed a high correlation with D type particles, showing strong fluorescence in the FL4 channel. The additional fluorescent data that were introduced also improved clustering attempts, making k-means clustering a comparable solution for this high-resolution data.

## 1. Introduction

Aerosols constitute a significant portion of the contents of Earth’s atmosphere. This ubiquity results in significant ecological, health and climatic effects on the planet. Aerosols are defined as suspensions of liquid or solid particles in a gas which possess a broad range of physical diameters; spanning the ultrafine (i.e., <100 nm) to the super coarse >10 mm [1,2,3,4]. PBAP make up a substantial fraction of the total aerosol and encompass particle types such as pollen, fungal spores and bacterial cells/agglomerates amongst many others [5].

The study of PBAP has recently undergone significant growth stemming from the ever-accumulating evidence that certain biological particles are linked to detrimental health conditions [6]. High concentrations of both fungal spores and pollen grains have been implicated in numerous diseases and health problems for humans, flora and fauna. For example, respiratory problems such as chronic obstructive pulmonary disease (COPD), allergic rhinitis, asthma and farmers’ lung have been conclusively linked to aeroallergen interactions within both humans and animals [7,8,9]. Several spore forming fungal species are also known to produce mycotoxins which has been linked with diseases such as cancer, as well as renal failure [10]. Many pollen and fungal spore taxa have also been shown to act as effective cloud condensation/ice nucleation particles [11,12,13,14,15,16,17,18], potentially impacting the climate, either directly through the scattering and absorbing of light or indirectly by influencing cloud formation and precipitation development [19].

Traditionally, PBAP numbers and types were determined by use of impaction instrumentation, whereby particles are sampled onto a suitable substrate before counting and identification. The analysis methods commonly adopted include optical microscopy or agar plate culturing [10]. However, these methods are incredibly laborious and time consuming and require trained operators to accurately identify specific types and species of pollen/fungal spores. More recently, the use of fluorescence detection in the real-time monitoring of ambient particles has grown in popularity. One such instrument is the WIBS. The WIBS detects and records airborne concentrations of Fluorescent Aerosol Particles (FAP) targeted for bioaerosol identification. To date, the WIBS has been utilised in several field [20,21,22,23,24,25,26,27] and laboratory [28,29,30,31,32,33] studies.

The WIBS has been deployed in a myriad of different environments to monitor ambient bioaerosol variations, including rainforest [34,35], urban [36], semi-rural [37], biowaste [38], green-waste [39], coastal [40], indoor [41] and clinical [42] environments. The popularity of these real-time instruments has surged for several reasons including: (a) superior time resolution (millisecond), (ii) the techniques are non-destructive and (iii) the processing requires few consumables for operation, and generally does not require an extensively trained operator to extract the data when compared to some of the more subjective conventional methods. Such monitoring devices have undertaken a continued evolution, adding increasing functionality and efficiency in detection of the particles of interest. Such an instrument was developed as an upgrade to the WIBS-4 and was termed the WIBS-4+ (the focus of this study).

The WIBS is based on the principle that the multiple excitation wavelengths can provide improved discrimination between bioaerosols types such as fungal spores and pollen [31,43,44]; however, this has not usually extended to sub-groups or individual species. Several laboratory studies have examined the fluorescent spectra of different pollen grains and found that the presence of chlorophyll-a could be used as a possible biomarker for grass and herb pollen due to unique fluorescence peaks at 670 nm [45,46,47]. To aid in the identification and differentiation between pollen types, a third fluorescence detector was incorporated to the WIBS-4 model (FL4, FL5) were introduced to detect chlorophyll-a fluorescence across the wavelength range of 600–750 nm. As a result, the system displayed two extra channels of fluorescence data (in addition to FL1, FL2 and FL3), termed FL4 and FL5 [48]. Although the standard WIBS-4 model covers the spectral emission range for chlorophyll-b, it cannot provide resolved spectral information to separate it from other fluorophores [49].

A previous proof of principle study was conducted with the WIBS-4+ [48] that illustrated the capability of the WIBS-4+ to provide real-time signals for pollen that tracked well with traditional methods. However, this study was conducted in an area with little diversity in PBAP. Therefore, examination of the successful differentiation of grass and herb pollen from other pollen types and fungal spores using the WIBS-4+ is required. 

Therefore, this research details the first field campaign using the WIBS-4+ instrument. The results of which compare the data obtained from the WIBS-4+ to that of the traditional Hirst–Lanzoni trap, for both fungal spore and pollen detection. The influence of various meteorological and environmental conditions on the sampling efficiency of the WIBS will be detailed in a second instalment.

## 2. Materials and Methods

### 2.1. Site

**Figure 1:**
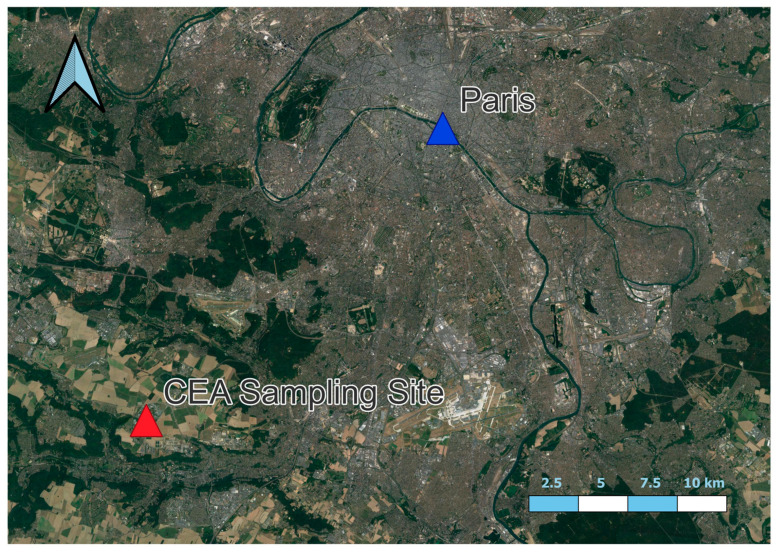
Site of the Bio detect 2 sampling campaign (Illustrating the proximity to the centre of Paris).

The WIBS-4+ was collocated with a Hirst–Lanzoni type bioaerosol impactor at the CEA in Scaly (48.7247° N, 2.1488° E) from 21/04/17 to 15/06/17. However, the WIBS was not in operation for 5 days from 05/05/17–10/05/17 due to power loss. Figure 1 illustrates the location of the site with respect to the major urban centre of Paris. Thus, the site can be considered a semi urban site given the surrounding countryside with the urban mega city of Paris 25 km to the north.

### 2.2. Hirst Instrumentation

Pollen and fungal spore data was collected using a Hirst–Lanzoni 7-day volumetric sampler [50]. The samplers drew in air at a rate of 10 L/min. Pollen and fungal spores present in the sampled air was then impacted upon a silicone-coated tape (Lanzoni) which rotates at a speed of 2 mm/h, thus completing one revolution every 7 days. On a weekly basis the tape was removed from the sampler, cut into daily segments, and mounted onto microscope slides. Each slide was counted manually using an optical light microscope at 400× magnification. Different pollen and fungal spore types were optically identified based on their morphological and structural features. The raw pollen and fungal spore counts was converted into daily concentrations (pollen grains/m^3^ and spores/m^3^) for each pollen/spore type.

### 2.3. WIBS Instrumentation and Data Acquisition

The WIBS is a single aerosol particle fluorescence monitor that uses light-induced fluorescence (LIF) to detect FAP and is commercially available from Droplet Measurement Technologies (DMT). However, this modified iteration of the WIBS was developed by the University of Hertfordshire, who originally developed the WIBS instrument [51,52]. The WIBS was originally designed for defence applications to enable the detection of airborne particles in locations where biological attacks might occur. It offers the ability to characterise the size and asymmetry (shape) of individual fluorescent and non-fluorescent particles by assessing the forward and sideways optical scatter, along with the spectrally unresolved fluorescent intensity of each particle at a millisecond time resolution. Several other publications have extensively discussed the internal workings of the WIBS instrument while thoroughly discussing the underpinning principles of its operation and potential application [29,30,53].

The major differences in this version of the WIBS and the aforementioned description is the addition of extra detection channels between 600 and 750 nm and a larger sizing range can be monitored (up to 30–40 µm). This was incorporated in attempts to visualise chlorophyll, which has been seen to be present in certain pollen grains, namely grass and weed pollen species [45,46,47].

Particles detected by the WIBS were designated fluorescent or non-fluorescent by comparing particle fluorescence intensity to a calculated baseline threshold. This threshold was determined by placing the WIBS into what is termed the forced trigger mode. This causes the instrument to fire on empty space (devoid of particles) for a predetermined time scale. The recorded fluorescence intensities following are then averaged and the standard deviation calculated. Following this the threshold for each fluorescent channel can be calculated as the mean intensity plus three times the standard deviation (3σ). During this campaign, the WIBS was placed into forced trigger mode daily to ensure no large variation was seen.

Upon determination of the fluorescent fraction, the particles were further categorised utilising Perring nomenclature [27]. Fluorescent particles were categorised into one of seven types, depending on the three forms of fluorescence signals detected by the WIBS [27]. These categories consider each channel individually (FL1, FL2 and FL3) but also include all possible combinations as shown in Table 1. Such cataloguing nomenclature allows for a more individual classification of each particle as previously particles could potentially be placed into one or more of the FL channels depending on the particle fluorescence. Thus, the designations used in Table 1 allow for a more detailed understanding of ambient particles fluorescent characteristics.

Due to the additional FL4 and FL5 channels incorporated into the standard WIBS-4 instrument, the WIBS-4+ classified fluorescent particles into three additional classifications: D, E and DE. Details summarising their excitation/emission wavelength criteria is also provided in Table 1.

### 2.4. K-Means

K-means clustering was performed using RStudio [54]. Raw data values were scaled prior to clustering. The k-means algorithm utilised FL1, FL2, FL3, FL4 and FL5 intensity, size and asymmetry factor (AF) to differentiate between different PBAP fractions. The ideal number of clusters was determined by calculating the Calinski-Harabasz index using the fpc package [55] within R.

## 3. Results and Discussion

### 3.1. An Overview of Pollen and Fungal Spore Concentrations Determined by the Hirst Instrument during the Monitoring Campaign

**Figure 2:**
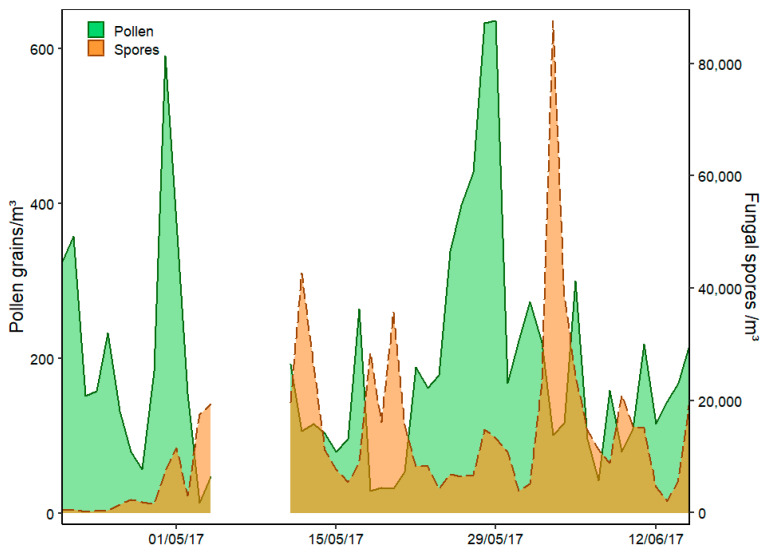
Time-series of daily pollen and fungal spore concentrations sampled by the Hirst with days with no WIBS data removed.

The ambient pollen and fungal spore concentrations were recorded by traditional Hirst volumetric method for the entirety of the monitoring period (21/04/2017–15/06/2017) and are presented below (Figure 2) as daily concentrations. In total, 47 pollen and 27 fungal spore types were identified. *Quercus* (28%), Poaceae (27%), Urticaceae (19%), *Pinus* (9%)*, Castanea* (3%)*,* Cupressaceae-Taxaceae (3%), *Sambucus* (2%)*, Rumex* (2%) and *Platanus* (1%) were the most prevalent pollen types identified. Whereas Ascospores (55%)*, Cladosporium* (31%), *Tilletiopsis* (4%), Basidiospores (3%) and *Helicomyces (3%)* represent the most dominant fungal spore types identified. The prevailing dominance of Ascospores and *Cladosporium* at the Saclay site is consistent with previously documented monitoring efforts [56].

During the monitoring period ambient pollen concentrations reached highs of ~600 grains/m^3^ on two separate occasions: initially on the 29th of April due to extremely high concentrations of *Quercus* pollen and again during the 28th–29th of May due to high Poaceae and Urticaceae concentrations. A smaller peak was also observed on the 11th of May attributed to high concentrations of *Pinus* pollen. In comparison, four separate fungal spore peak periods of greater than 30,000 spores/m^3^ were observed during the sampling period. The first peak on the 12th of May was caused by high concentrations of both Ascospores and *Cladosporium* spores. The next consecutive peak days on the 18th and 20th of May resulted predominantly from high concentrations of Ascospores and lastly, the highest daily fungal spore concentration was recorded on the 3rd of June equating to nearly 85,000 spores/m^3^, with Ascospores contributing to over 70% of the total daily concentration.

More detailed information regarding the interannual, seasonal and daily variability of total pollen grain, and fungal spore concentrations at Saclay has been covered thoroughly in two previous investigations [56,57]. The scope of both studies encompasses 4–5 years of monitoring data including the 2017 period investigated herein. In addition, ambient bacterial concentrations have also been monitored recently in Saclay [58].

**Figure 3:**
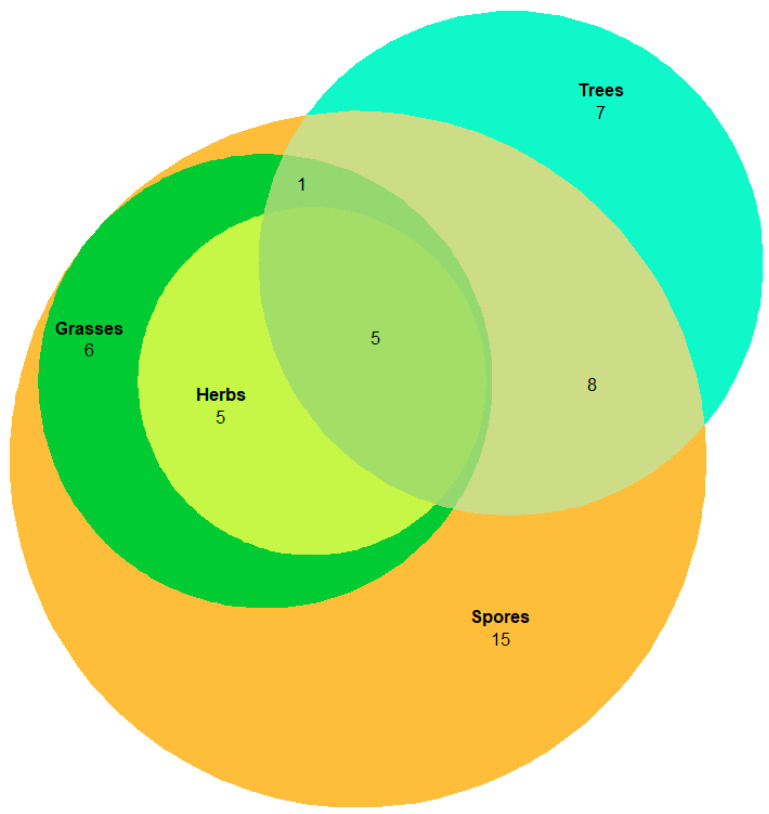
Euler diagram depicting days of co-occurring high concentrations of PBAP.

The co-occurrence of days with high pollen and spore concentrations are illustrated in Figure 3, below. The concentration levels denoted as “High” were established in accordance with literature values or based on their major constituent species/families. Days of high grass concentration were classified as days exceeding 50 grains/m^3^ [59]. In the case of herb pollen, which was vastly dominated by Urticaceae, days were classified as “high” if daily concentrations exceeded 80 grains/m^3^. Concentrations above this level have been previously shown to instigate health reactions when exposed to allergenic species within the Urticaceae family [60]. In the case of tree pollen, threshold values were based on limits previously introduced for Quercus pollen, which dominated the tree pollen fraction recorded in Saclay. High levels of tree pollen were classified as days exceeding 80 grains/m^3^ [61]. In the case of fungal spores, days exceeding 2500 spores/m^3^ were classed as “high”. Due to the lack of recommended literature values on total Ascospores, this limit is largely based on *Cladosporium* spores concentrations [59,62,63] which has been shown to possess higher seasonal background levels than other spore types [64].

From examination of Figure 3, there are 5 days during which high concentrations of fungal spores, grass, herb, and tree pollen were evident. There were numerous other occasions when overlapping high concentrations of the different PBAPs were also observed. From a monitoring point of view, any real-time detection method should be able to differentiate between these major PBAP classes.

### 3.2. An Overview of Fluorescent Particles Detected by WIBS-4+ during the Monitoring Campaign

Simultaneous fluorescent particle monitoring was carried out by the WIBS-4+. The WIBS classifies particles as either fluorescent or non-fluorescent based on a predetermined threshold (3σ). The majority of particles sampled over the course of the campaign were seen to be non-fluorescent (62%). Hence, approximately 38% of particles were classified as fluorescent.

This fluorescent fraction of the total aerosols were further classified into one of seven traditional particle types [27]. The fluorescent fraction was also classified separately according to their intensity in the FL4 and FL5 channels (D, E, DE). Contributions to each classification are summarised in Table 2.

Whereas AB, B, ABC, A and BC type particles contribute considerably to the fluorescent fraction of particles, AC and C particles were seen to contribute negligibly (<0.5 %). The striking absence of these particle types, especially AC, has been well documented during other WIBS campaigns and laboratory studies [27,65]. Of all the particle classes observed, AB particles were the most dominant. Traditionally, his fraction has been identified as being bacterial or fungal in origin [53,65]. Several of the other classes, namely ABC and A type particles have also been shown to originate from biological sources (pollen and fungal/bacterial sources, respectively) [65,66].

Interestingly, B type particles were seen to be the second most prevalent particle type. This particle fraction has been largely linked to anthropogenic sources [31,36,67], and somewhat to riboflavin and other minor PBAP components [53]. However, due to the minor contribution of B particles to bioaerosol fractions, it has been recommended that any atmospheric sampling in which type B is a dominant fluorescent type should be examined carefully for potential interferents [65]. Saclay is a semi urban location that could easily be subject to various anthropogenic emissions, especially when considering its proximity to the megacity, Paris. This could explain the high concentrations of B particles observed.

**Figure 4:**
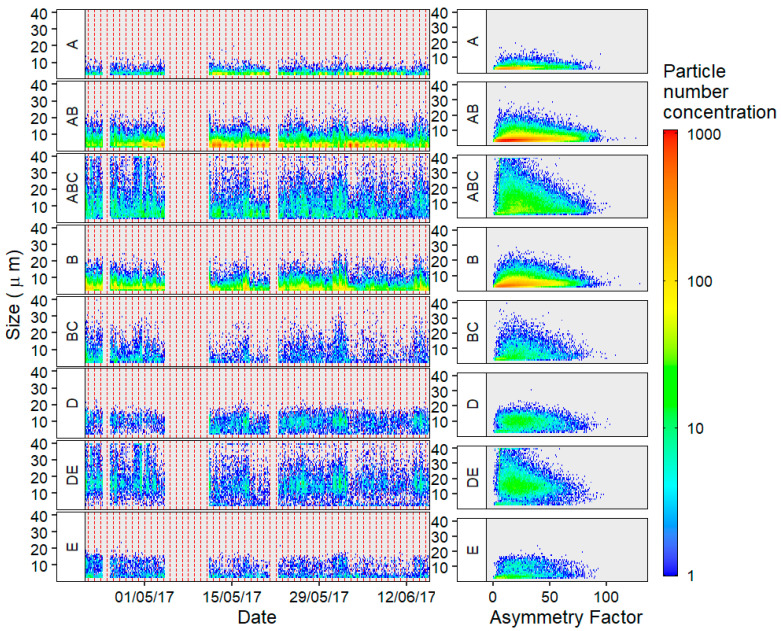
Daily temporal trends (left—where each area within the red dotted line illustrates 1 day) and Size vs. Asymmetry Factor distribution (right) of the largest contributors to the fluorescent particle trends.

The size and AF distributions of the prevalent fluorescent fractions were also examined (Figure 4). Comparing the AF and size distributions of each particle type can illustrate the variance within each particle category. ABC type particles were seen to peak at larger size ranges with a median AF value of 25, which could be indicative of pollen-like particles. This would corroborate previous studies which found that particles with high FL2 and FL3 fluorescence intensities, larger size and lower AF values tended to correlate well with pollen [30].

AB and A type particles were seen to contain a high concentration of small particles of <10 μm with median AF values of 23 and 18. Low AF values such as these are indicative of spherical particles that could be fungal in nature [35]. However, the broad spread of AF distribution for AB particles may represent higher contributions of more off-spherical fungal spore morphologies [30].

**Figure 5:**
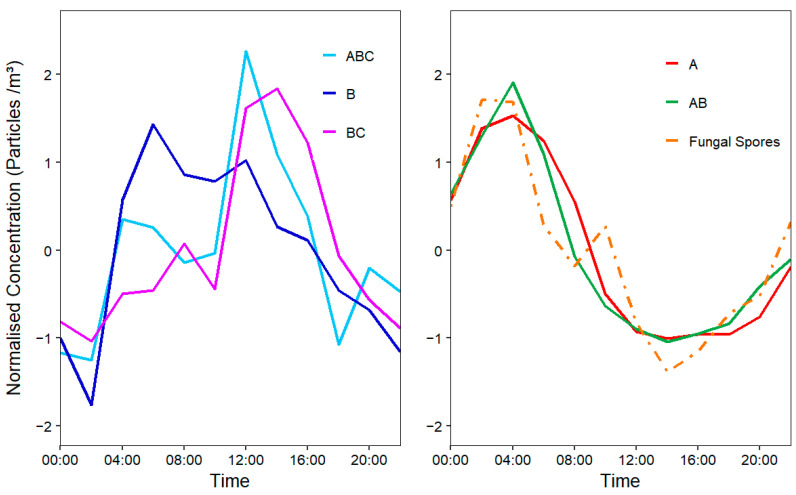
Normalised Diurnal (local time) fungal spore concentrations sampled via the Lanzoni compared to isolated WIBS particle concentrations.

Examination of the average diurnal trends of the classified WIBS classes provided additional information on the possible contributing PBAP fractions. As seen in Figure 5, the average hourly trends of the A and AB particles were seen to peak in the early morning (04:00–06:00) and to continually rise again from late afternoon (16:00) onwards. Similar trends have been noted in literature for fungal spores [21,38]. This gains further credence when the total fungal spore diurnal trend is compared. The average daily trend in ambient fungal spore concentration sampled by the Hirst is seen to mirror the trends seen in A and AB particles. In comparison, contrasting trends were noted for B, BC and ABC types with peaks occurring during the middle of the day.

Utilising the additional FL4 and FL5 channels, fluorescent particles were separately classified into D, E and DE classes. From examination of Figure 4, it is noted that they are less abundant than the other FAP classes—at approximately one tenth the concentration. These classes also possess a more uniform distribution in particle size and AF. They are less impacted by smaller size ranges; this is particularly true for DE type particles, which exhibited a median particle size of 15 µm and AF of 23 which could also be representative of pollen-like particles.

### 3.3. Hirst vs. WIBS-4+

The FAP data obtained from the WIBS was used to construct daily profiles that were comparable to both fungal and pollen concentrations recorded by the Hirst. However, comparing the WIBS to the Hirst is not without difficulty since both systems utilise vastly different operating principles. As highlighted by previous investigations, any particles too large or too small for efficient collection by the instrument will be undercounted [21]. For any fluorescence-based instrument, it has also been speculated that some bioaerosols may fluoresce too weakly to be detected in certain circumstances [21].

The WIBS operates at a considerably higher resolution but lower flow rate than the Hirst, giving it a much higher capability for sampling smaller particles. The WIBS can record particles as low as 0.5 μm in size, making it applicable for monitoring other small bioaerosols such as bacteria. Whereas the Hirst is limited by its use of microscopic analysis and is heavily dependent on the operator. As such the Hirst is less efficient at monitoring particles smaller than ~2 μm in size [22]. As a result, differing numbers of FAP and fungal spores/pollen grains were sampled by the WIBS and Hirst. To aid in comparing the two methods, WIBS particles less than 2 µm were removed from the collected fluorescent data prior to analysis.

#### 3.3.1. Comparison of Fungal Spore Concentration with Fluorescent Particles (WIBS)

A direct analysis of fungal spore vs. FAPs concentrations was undertaken in an attempt to further scrutinise the proposed link between fungal spores counted via the direct impaction device. Initial correlation analysis (Figure S1) was carried out, comparing daily fungal spore concentrations with the different FAP classes. Good correlations between fungal spores and A/AB were observed returning Pearson correlation coefficients of 0.81 for total fungal spores. Equally these AB and A types were seen to correlate well with each other. Alluding to the possibility that both are related to fungal spore concentrations, corroborating previous observations [30,53,65]. Negative correlations were noted for B and BC types with C and ABC types displaying no significant correlation. The lack of correlation with ABC particles is worth noting as they have been somewhat associated with fungal spore counts in previous studies [22,26,68]. Upon inclusion of the additional D, E and DE classes, a negative correlation was observed for fungal spores and DE and no relationship was observed for D and E type particles. A similar lack of correlation was also observed for A and AB particles when compared to the new FL4 and FL5 classes, further suggesting the potential fungal characteristics.

This notable negative/lack of correlation seen between fungal spores and FL4/FL5 fluorescence could, partly be attributed to the fact that fungal spores do not contain chlorophyll. Although, the excitation and emission bands used for these channels were included in efforts to target the fluorescence observed for certain pollen types with the 600–750 nm range attributed to the presence of chlorophyll. There are numerous other compounds that could also induce fluorescence in these channels that could be present in different PBAP. These could include a number of constituents of sporopollenin such as phenolics, carotenoids, anthocyanin, azulene as well as chlorophyll [69,70]. The observed correlation between fungal spores and D, DE and E particles are, therefore, not exclusively related to the lack of chlorophyll but rather the lack of specific fluorescent compounds.

Greater linear correlation was found for fungal spore concentrations when the A and AB particle categories were combined. The trend of A+AB particles was seen to closely mimic that of the observed fungal spore concentration, producing an R^2^ value of approximately 0.7. Taking the two dominant fungal spore types into account, it was noted that Ascospores followed similar trends to A+AB particles less than 5 µm in size (r = 0.83), whereas *Cladosporium* concentrations were seen to resemble A particle trends less than 10 µm in size (r = 0.66). Although good agreement was seen for *Cladosporium* spores and A particles, a notable decrease in correlation is apparent when compared to the performance of Ascospores. The WIBS has shown an inability to fully characterise sharp increases in *Cladosporium* concentrations [22]. Several reasons have been disputed for this observation, from the physical aspects of *Cladosporium* (it can be released in clusters) meaning the WIBS can misidentify such clusters as one larger particle, these clusters leading to increased wall loss, to photophysical properties of the fungal spore inhibiting the absorption of light by bio fluorophores within the cell [21,71]. As a result, these concerns could be contributing to this slight reduction in *Cladosporium* sensitivity.

However, several interferents can also contribute to the fluorescent particle signals monitored by the WIBS. Natural processes can affect the measured fluorescence of bioaerosols. Atmospheric ageing and water uptake has been shown to directly influence the fluorescence signal and intensity of bioaerosols [36,72]. Moreover, many studies have particularly highlighted the potential interference caused by anthropogenic aerosols. These include polycyclic aromatic hydrocarbons (PAH), humic-like substances (HULIS), mineral dust, secondary organic aerosols and black carbon [53,69,73]. These substances not of biological origin, have been shown in previous studies to contribute to the fluorescence signals. In efforts to limit the effect of potentially interfering aerosols, the initial fluorescent threshold was increased from 3σ to 6σ and 9σ. The original fluorescent threshold is increased to 6σ and 9σ by increasing the magnitude of standard deviations added to the mean fluorescent intensity. Whereas the 3σ threshold was determined by the addition of three standard deviations to the mean fluorescence intensity of each channel (when the instrument is sampling in forced trigger mode); 6σ and 9σ refers to the addition of six and nine standard deviations to the mean fluorescence intensity, respectively. Increasing the fluorescent threshold in this manner has been shown to significantly reduce the interference from non-biological aerosols but not affect the relative fraction of bioaerosols detected by the WIBS [53].

**Figure 6:**
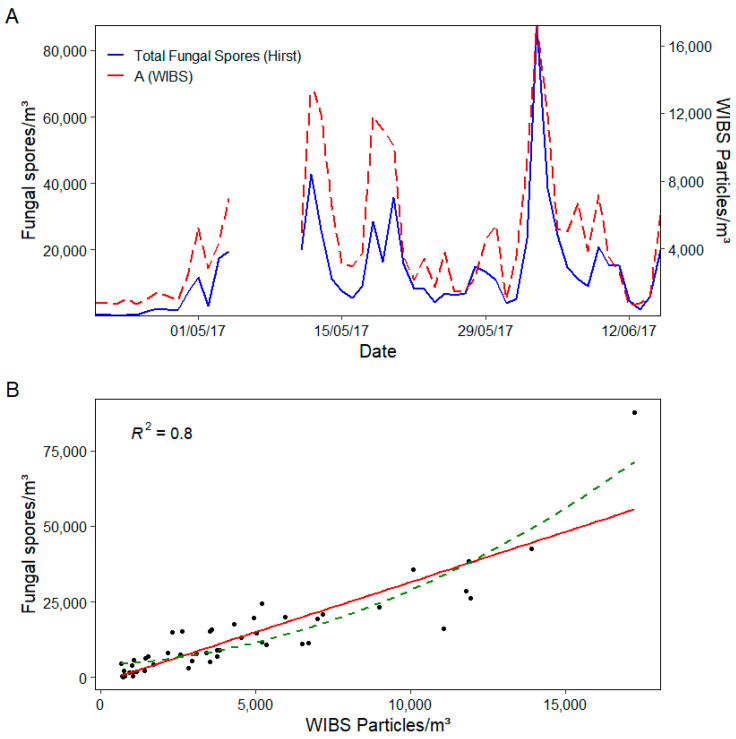
(**A**) Daily time series of Hirst fungal spore vs. WIBS A (9σ) particle concentrations and (**B**) scatter plot of the relationship between daily Hirst fungal spore and WIBS A particle concentrations (depicted as black dots), where the red line represents a linear regression, and the green line represents a polynomial regression.

By doing so, a marked improvement was noted for the correlation between A type fluorescent particles and total fungal spore concentrations, as illustrated in Figure 6 below.

The R^2^ for total fungal spores increased from 0.7 (3σ) to 0.8 (9σ). It is worth highlighting that although the R^2^ illustrated in Figure 6B is based on the linear regression of the relationship between total fungal spores and A particles, the polynomial regression (green line) gives a slightly higher R^2^ value of 0.84. By increasing the fluorescent threshold, many remaining AB type particles were shifted to the A type category, thus giving similar results to the originally combined A and AB particle fraction with the presumed removal of interfering components. Any remaining interferents are likely to be very highly fluorescent anthropogenic or biological aerosols. For example, bacteria have also shown to contribute significantly to A particle fractions [65] and although many are removed as a result of size filtering the FAP (<2 µm), larger bacteria of up to 5µm that would not be counted microscopically could be contributing to the A particle fraction at these higher fluorescent thresholds.

#### 3.3.2. Comparison of Pollen Concentration with Fluorescent Particles (WIBS)

Following initial correlation analysis (Figure S1), total pollen concentrations were found to correlate strongest with DE particles (r = 0.48), with ABC (r = 0.32), B (r = 0.41) and BC (r = 0.28) type particles also possessing notable correlation. Both ABC and BC particle classes have been shown previously to be indicative of pollen grains [53,65]. Considering the higher particle size ranges observed for these particle classes, it is possible both could be related to pollen grains. Following further size filtering, ABC particles greater than 15 μm illustrated the strongest correlation with total pollen yielding a Pearson correlation (r) of 0.5.

**Figure 7:**
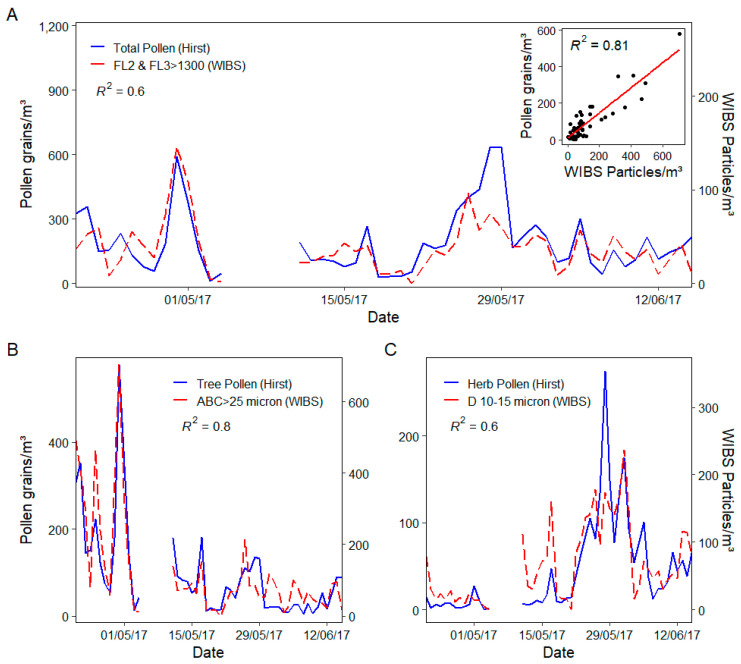
(**A**) Daily time series and embedded linear regression of Hirst total pollen vs. WIBS Particle concentrations with FL2 and FL3 intensity greater than 1300, (**B**) Daily time series of Hirst tree pollen vs. WIBS ABC (greater than 25 µm) particle concentrations and (**C**) Daily time series of Hirst herb pollen vs. WIBS D (10–15 µm) particle concentrations.

Previous WIBS campaigns have concluded that although ambient pollen grains possess high fluorescence intensity in all 3 channels, they are most fluorescent in the FL2 and FL3 channels [20]. Therefore, in efforts to more accurately isolate pollen type FAP a range of size and FL2/FL3 filters were applied to WIBS data. The most representative conditions for total pollen involved isolating particles larger than 2 μm with FL2 and FL3 fluorescence greater than 1300 which equated to an R^2^ of 0.6, as illustrated in Figure 7. From examination of the regression scatter plot, two outliers that significantly affect the plot can be observed, upon their removal a stronger association (R^2^ = 0.7) is observed.

Overall, the isolated FAPs follow the trends of the observed total pollen concentrations for the majority of the monitoring campaign but fail to account successfully for the peaks during the 11th (*Pinus* pollen) and 28th–29th (Poaceae pollen) of May. Of the pollen types recorded, both *Pinus* and Poaceae would be considered to have some of the largest pollen grains. The WIBS has shown an increased efficiency for sampling smaller particles [33] and has difficulty in efficiently detecting large pollen types due to the relatively low flow rate used and is often under sampled [20]. As such, similar comparison studies often observe lower “pollen” concentrations being recorded by the WIBS in comparison to the Hirst [37]. Larger particles such as Poaceae and other larger pollen types can also experience unavoidable sedimentation losses in the system inlet, further contributing to under sampling [40,49,74,75]. This likely resulted in the observed under sampling of total pollen concentrations illustrated in Figure 7.

While the determination of total pollen concentrations is of interest, more often than not specific pollen types can be considered more “important” than others due to their relative allergenicity [57]. For the Saclay region, five tree species belonging to the Betulaceae and Oleaceae families, two shrub families (Cupressaceae-Taxaceae) and two herbaceous families (Poaceae and Urticaceae) are considered to be of high importance due to their allergenic prevalence and health impacts [8,57,76]. Therefore, to evaluate the potential of using FL4 and FL5 channels in differentiating between different pollen types, pollen taxa identified by the Hirst were categorised as “grasses”, “herbs” and “trees and shrubs (trees)”. As was the case with fungal spore analysis, the original fluorescent baseline threshold (3σ) was increased to 6σ and 9σ to limit the effect of potential fluorescent interferents. Although, many chemical interferents are likely to be smaller in size than pollen grains, several have been shown to generate fluorescent aerosols of up to 10 µm in size. This is particularly true for soil dust which is shown to favour coarser size modes and soot particles, which can form to be as large as some pollen grains [36].

Grass pollen showed reasonable correlation with D type particles greater than 10 µm (R^2^ = 0.4), which exceeds any correlation observed for any of the original 7 WIBS particle types. Once again deviations from the expected pollen trends were apparent during periods of high grass pollen concentration due to the under sampling of Poaceae pollen by the WIBS. Similarly, herb pollen showed a considerable correlation with D type particles within the size range of 10–15 µm (R^2^ = 0.6) as illustrated in Figure 7, such size ranges are indicative of the herb pollen types such as Urticaceae [30]. A clear relationship between the two instruments for daily herb concentration can be seen. However, at times of higher herb pollen concentration measured by optical microscopy, deviations were observed for the corresponding fluorescence signals recorded by the WIBS-4+. Such deviations could be elucidated due to differences in instrument operating principles. The Hirst in particular has been used extensively since the 1950s, despite its historical popularity, there are a number of drawbacks in using the Hirst that can lead to deviances when compared to real-time devices [77]. For one, the PBAP concentrations determined by the Hirst method are based on the statistical extrapolation of a portion of the counted sample slide. This can result in large errors of up to 30% and thus can potentially provide somewhat biased ambient concentrations [78] leading to notable differences when compared to real-time devices. As previously mentioned, the enhanced flow rate of the Hirst makes it more efficient at sampling larger (potentially faster moving) particles that are unaffected and thus under sampled by the lower flow rate of the WIBS. Meteorological conditions such as strong wind speeds could also influence the sampling efficiency of the instruments [77,79]. By design the Hirst sampler continually orientates itself to face the prevailing wind. This means it can overestimate pollen and fungal spore concentrations when compared to more isokinetic samplers such as the WIBS [80,81], This could explain why certain pollen peak periods were not witnessed by the WIBS. The effects of ambient conditions such as meteorological factors and anthropogenic emission on the operation of the WIBS-4+ will be further examined and detailed in a second publication.

Tree pollen has previously shown to be efficiently detected by the WIBS. The WIBS-4 model was able to successfully detect Yew pollen yielding an R^2^ value of 0.9 [20]. Such proof-of-principal studies have shown the capability of the WIBS to monitor select pollen species in less diverse environments but have stated that the introduction of other PBAP types such as additional pollen taxa and fungal spores could further complicate the selective monitoring ability of the WIBS. However, in this case, daily concentration comparisons of both the Hirst and the WIBS were in full agreement for tree and shrub pollen as shown in Figure 7. This relationship was most apparent for ABC particles greater than 25 µm, showing excellent daily correlation with a computed R^2^ value of 0.8. This size range is representative of the dominant tree species observed during the sampling period and are considered large pollen grains with average sizes exceeding 25 µm, indicating that the WIBS-4+ designation of “size” would appear to be consistent with the physical diameter evaluated optically [20]. *Quercus* pollen alone was the most dominant pollen witnessed during the monitoring period, representing almost a third of all pollen observed and has previously shown to correlate well with FAP greater than 25 µm in size [20]. A significant deviation was observed for tree and shrub pollen and ABC for the 11th of May, this was attributed to high concentrations of *Pinus* pollen, likely under sampled by the WIBS. Upon removal of *Pinus* pollen from the total tree and shrub pollen concentration, a slight increased correlation (increase in r from 0.88 to 0.92) was observed. Therefore, to selectively monitor pollen routinely using the WIBS-4+, modifications to ensure the effective and representative sampling of a range of pollen sizes may be necessary. This is particularly true for larger pollen grains less affected by the current WIBS flow rate.

**Figure 8:**
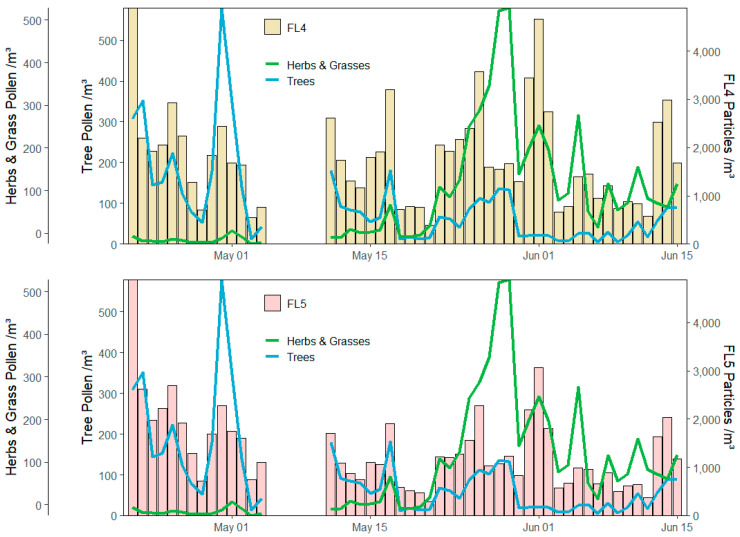
Daily distributions of select pollen classes and FL4/FL5 fluorescent particles.

From the additional particle classes introduced in this investigation (D, E, DE), tree and shrub pollen showed good correlation with DE type particles, illustrating clear differentiation from the herb and grass pollen. This correlation with the newly introduced FL4 and FL5 channels can be seen in Figure 8. Although it is expected particles illustrating fluorescence in the FL4 and FL5 channels would follow the occurrence of grass and herb pollen due to the presence of chlorophyll, considerable correlation was also witnessed for tree pollen. This was particularly apparent during the early half of the campaign during which, concentrations of *Quercus* pollen were high. This relationship between tree pollen and DE particles was only marginally outperformed by ABC type particles. Although tree pollen is known to lack the fluorescence corresponding to chlorophyll, there are other compounds such as terpenoids and azulenes that are present in tree and shrub pollen that can exhibit fluorescence in the 600–750 nm region [46,70,82]. The presence of these fluorescent compounds has resulted in tree pollen also being classified as DE type particles. For more selective identification of chlorophyll-a, sharper detection of emission at ~670–680 nm would likely be required.

### 3.4. K-Means Clustering

Other methods such as cluster analysis [83,84,85] have also exhibited the capability to differentiate between FAPs. In this case, K-means clustering was employed to explore more practical methods of identifying bioaerosol fractions from the WIBS data. K-means clustering has been applied to WIBS data in the past but has often been outperformed by more computationally taxing unsupervised methods such as Hierarchical clustering [66,83,84], and more recently, supervised methods such as gradient boosting [85]. These methods require advanced computational resources and often the use of supercomputers due the extremely high-resolution datasets produced by the WIBS. The analysis of such data likely represents the time-limiting step for the real-time deployment of the WIBS, especially if the required computational power is not available/affordable. K-means requires less processing time and power compared to other clustering methods, making it a desirable alternative. Previous studies have disregarded the potential of k-means clustering for WIBS data analysis due to the production of similarly sized groups [84]. However, the method has recently shown potential in differentiation several pollen taxa using data obtained from another fluorescence based bioaerosol sensor [86].

For this investigation, the unsupervised K-means method yielded representative clusters of both pollen and fungal spore concentrations from the WIBS particle data. K-means clustering was carried out on all fluorescent WIBS particles including FL4 and FL5 fluorescence variables. The inclusion of FL4 and FL5 fluorescence values improved the differentiation between the pollen and fungal spore clusters. Ten clusters were identified with Cluster 7 demonstrating a good relationship with pollen concentrations, with an R^2^ of 0.55. Similarly, Clusters 1 and 5 correlated significantly well with fungal spores, producing a combined R^2^ of 0.63.

**Figure 9:**
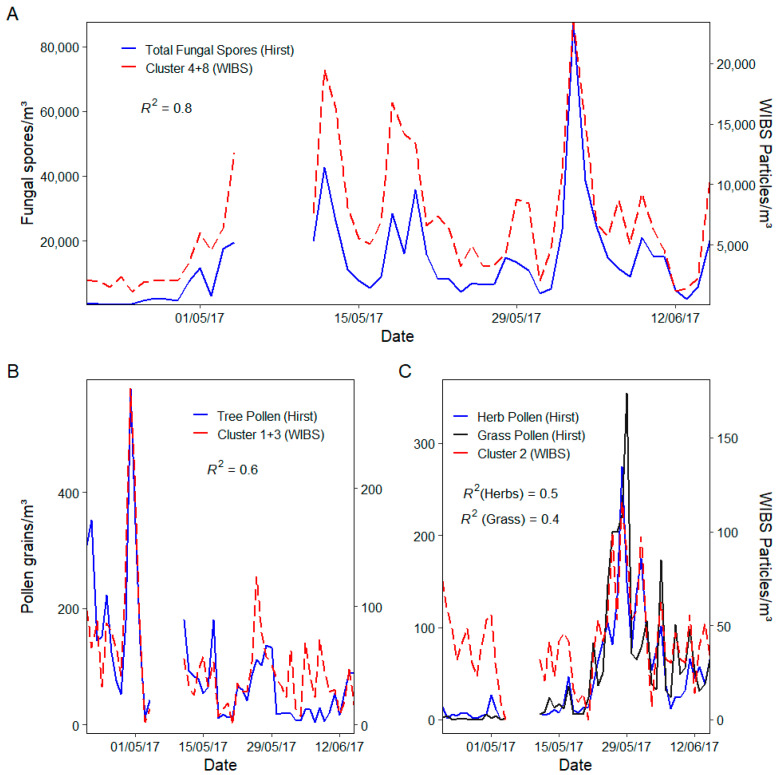
(**A**) Daily time series of Hirst fungal spore concentrations vs. resulting WIBS K-means clusters (4 + 8), (**B**) Daily time series of Hirst tree pollen concentrations vs. resulting WIBS K-means clusters (1 + 3) and (**C**) Daily time series of Hirst grass/herb pollen concentrations vs. resulting WIBS K-means clusters (2).

K-means was also used to further improve the relationship of the original particle combination of A and AB (3σ) for fungal spores. Utilising this intra-categorical k-means clustering allowed for further filtering of A+AB type particles, yielding clusters that could be more representative of fungal spore concentrations than the simple sum of all A+AB particles. Nine clusters were determined from the analysis of all A and AB particles. Cluster 4 and 8 showed the highest association with fungal spores, yielding an R^2^ value of 0.8 (Figure 9). Clusters 4 and 8 also exhibited representative size ranges of between 2–7 µm and 2–8 µm, respectively.

The same k-means approach was also applied to WIBS particles with FL2 and FL3 fluorescence intensity greater than 1300 which has been shown to possess correlation with total pollen, providing an original R^2^ of 0.5. K-means clustering yielded 3 clusters. Interestingly, the clusters showed good differentiation between the different pollen types. Cluster 2 showed notable correlation with the dominant grass and herb pollen types equating to R^2^ values of 0.4 and 0.5, respectively. Cluster 2 particles were found to be between 2–30 µm, with the median value of 17 µm, which could be indicative of the presence of Urticaceae pollen. Deviations from the expected pollen count were noted for periods of high concentration attributed to differences in instrument sampling efficiencies as discussed previously. Similarly, clusters 1 and 3 showed a strong equivalence to tree and shrub pollen yielding an R^2^ of 0.6 (Figure 9). Cluster 3 particles possessed a median size of 22 µm and showed a significantly strong association with *Quercus* pollen (R^2^ = 0.61) which represented ~30% of all the pollen sampled during the campaign.

Overall, the k-means clustering approach showed comparable results to those achieved for filtering of the 9 original FAP groups discussed above. The inclusion of FL4 and FL5 channels provided additional dimensions that distinguished FAPS of difference origins, potentially making k-means clustering a more suitable method for WIBS data analysis than was previously considered. K-means clustering might offer a time effective alternative that still provides good differentiation between pollen and fungal spore concentrations, providing the addition of FL4 and FL5 channels.

## 4. Conclusions

This study represents the first field campaign for the deployment of the WIBS-4+. Isolated WIBS FAPs correlated well with both ambient total pollen (R^2^ = 0.6) and total fungal spore (R^2^ = 0.8) concentrations. The WIBS-4+ utilises the original three WIBS fluorescent channels with the addition of two further channels used to target the presence of various pollen specific components such as chlorophyll, which has previously been shown to distinguish grass/herb pollen from other pollen and PBAP types. In addition to chlorophyll, several other sporopollenin components presumably also fluoresce in the FL4 and FL5 detection bands. The detection of these sporopollenin components showed promise in selectively identifying pollen grain from fungal spores. This also led to grass and herb pollen illustrating good correlation with D type particles and DE particles illustrating a strong agreement with tree and shrub pollen concentrations.

Overall, the inclusion of the additional detection bands improved differentiation between tree/shrub (R^2^ = 0.8), herb (R^2^ = 0.7) and grass pollen (R^2^ = 0.4) and improved K-means clustering attempts for both pollen and fungal spores. Although the FL4 and FL5 channels are capable of distinguishing broad classes of bioaerosols such as pollen, from other biological and interfering aerosols, the relative sizes of certain pollen types led them to being under sampled by the WIBS. Future iterations of the WIBS designed for aerobiological purposes will likely require not only the additional fluorescent detection bands but also modified sample flow. The strong agreement seen between the FL4 and FL5 FAPs and ambient pollen concentrations also might indicate that using these excitation/emission bands alone in the development of future sensors, could hold some promise for the detection of certain bioaerosols such as pollen.

Additional research will also be undertaken to further assess the correlation between air quality and meteorological parameters on the bioaerosol monitoring conducted (WIBS-4+ and Hirst) during this study.

## Figures and Tables

**Table 1:** WIBS channel annotation matrix. Channels are matched with excitation wavelength and emission waveband.

Channel	Excitation (nm)	Emission (nm)
A	280	310–400
B	280	420–650
C	370	420–650
AB	280	310–400
		420–650
AC	280	310–400
	370	420–650
BC	280	420–650
	370	
ABC	280	310–400
		420–650
	370	420–650
D	280	600–750
E	370	600–750
DE	280	600–750
	370	

**Table 2:** WIBS particle distribution (% of total fluorescent particles) using Perring nomenclature.

Particle Class	Percentage Contribution
AB	49%
B	30%
ABC	10%
A	7%
BC	3%
C	<0.5%
AC	<0.5%

## Data Availability

Not applicable.

## Supplementary Materials

The following supporting information can be downloaded at: https://www.mdpi.com/article/10.3390/s22228747/s1, Figure S1: Correlation table showing the Pearson correlation between different fluorescent particle types and different PBAP classes. The colour bar and circle size indicate positive and negative correlation, with larger circles and more intense colour larger sizes.
