# Supplementary material for: A Modified Spectroscopic Approach for the Real-Time Detection of Pollen and Fungal Spores at a Semi-Urban Site Using the WIBS-4+, Part I
Source: Sensors (Basel). 2022 Nov 12;22(22):8747. doi: 10.3390/s22228747 (PMC9694534; doi:10.3390/s22228747)

Figure S1: Correlation table showing the Pearson correlation between different fluorescent particle types and different PBAP classes. The colour bar and circle size indicate positive and negative correlation, with larger circles and more intense colour larger sizes.

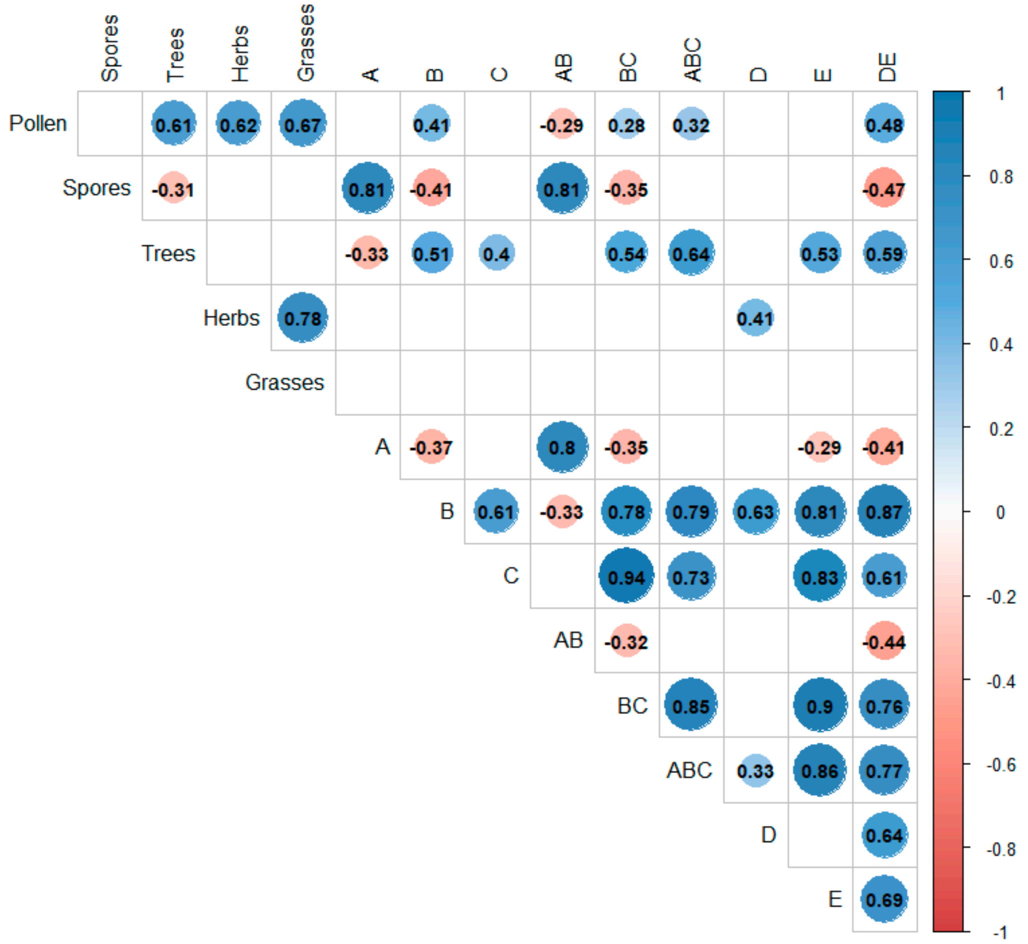

Supplement: Supplementary file 1 [file sensors-22-08747-s001.zip › sensors-1993847-supplementary.pdf]
